# Climate change-induced heat risks for migrant populations working at brick kilns in India: a transdisciplinary approach

**DOI:** 10.1007/s00484-017-1476-0

**Published:** 2017-11-30

**Authors:** Karin Lundgren-Kownacki, Siri M. Kjellberg, Pernille Gooch, Marwa Dabaieh, Latha Anandh, Vidhya Venugopal

**Affiliations:** 1 Department of Design Sciences, Lund University, 221 00 Lund, Sweden; 2 Department of Human Geography, Lund University, Lund, Sweden; 3 Department of Architecture, The British University, Cairo, Egypt; 4 Department of Environmental Health Engineering, Sri Ramachandra University, Chennai, India; 5 Academy of Maritime Education and Training (AMET) University, Chennai, India

**Keywords:** Brick kilns, Climate change, Heat stress, India, Migrant work, Technical and socio-cultural solutions, Transdisciplinary approach

## Abstract

During the summer of 2015, India was hit by a scorching heat wave that melted pavements in Delhi and caused thousands of deaths, mainly among the most marginalized populations. One such group facing growing heat risks from both occupational and meteorological causes are migrant brick kiln workers. This study evaluates both current heat risks and the potential future impacts of heat caused by climate change, for the people working at brick kilns in India. A case study of heat stress faced by people working at brick kilns near Chennai, India, is the anchor point around which a transdisciplinary approach was applied. Around Chennai, the situation is alarming since occupational heat exposure in the hot season from March to July is already at the upper limits of what humans can tolerate before risking serious impairment. The aim of the study was to identify new pathways for change and soft solutions by both reframing the problem and expanding the solution space being considered in order to improve the quality of life for the migrant populations at the brick kilns. Technical solutions evaluated include the use of sun-dried mud bricks and other locally “appropriate technologies” that could mitigate the worsening of climate change-induced heat. Socio-cultural solutions discussed for empowering the people who work at the brick kilns include participatory approaches such as open re-localization, and rights-based approaches including the environmental sustainability and the human rights-based approach framework. Our analysis suggests that an integrative, transdisciplinary approach could incorporate a more holistic range of technical and socio-culturally informed solutions in order to protect the health of people threatened by India’s brick kiln industry.

## Introduction

India is experiencing increasing heat both in the economy and in rising temperatures (IPCC 2013). The people suffering the most from actual heat, such as rural populations and migrant daily wage laborers, are to a large degree left out of by globalization and neoliberal capitalism while still bearing the brunt of its negative consequences (Klein 2009; O’Brien et al. 2004). The structural adjustment programmes (SAPs) launched in the early 1990s led India into a new economic era and transnational corporation export-oriented growth that has mainly manifested in its cities, widening the inequality gap between rural and urban populations. One consequence of this is a booming building industry that drives demand for clay bricks from brick kilns that employ rural migrant labor (Kumbhar et al. 2014). Here, we find two sides of India emerging as the paradox of a building technology shaping the new while still based on ancient techniques of fired soil and human labor (SDC 2008). Hence, at one extreme, new high-rise buildings are hosting busy modern life of people from India’s growing middle class, working for global companies and cooled down by air conditioning. At the same time, India’s local economy long nurtured by rural village farming populations found themselves falling into increasingly precarious circumstances, forced off the land, often due to mounting debts, and into the migrant working force with many ending up at brick kilns. Generally, the most vulnerable populations, such as migrant workers and the elderly, have borne the unjust burden of climate change consequences (Klein 2009; NDMA Government of India 2016; UNDP 2016).

The negative impact of the SAPs on agricultural policies for rural labor, combined with climate change-driven factors that have worsened the dry season, has forced rural populations and landless migrant workers to travel from the poorest of Indian states, such as Bihar, Orissa, Uttar Pradesh, and West Bengal, into low-tech jobs, such as brick making. The brick kiln sector in India employs millions of migrant workers (SDC 2008). Though now illegal in India, various forms of bonded labor that amount to essentially forms of slavery, including child labor, continue to be common practice throughout India’s brick industry due to weak law enforcement (Guérin et al. 2012). Families, including young children, work in harsh, low-paying conditions, commonly compensated piece by piece (Khandelwal 2012). There is typically a lack of basic facilities, such as access to clean drinking water and sanitation (Inbaraj et al. 2013). Risks to health are many and the sector has high death rates. Health impacts mainly originate from breathing in smoke and physically demanding work outdoors combined with extreme weather causing heat strain and other illnesses such as pneumonia and respiratory infections (Nizami 2011; Pingle 2012). Long-term brick kiln workers, who adopt a specific posture for prolonged working periods, commonly develop severe musculoskeletal problems (Inbaraj et al. 2013).

The aim of the study was to identify new pathways for change and soft solutions by both reframing the problem and expanding the solution space. The objective was to look at the problems faced at brick kilns from different angles, using a transdisciplinary approach to identify soft solutions for migrant brick kiln workers in Chennai. This approach is novel in the current brick kiln literature that has otherwise predominantly looked at technical aspects. The intended audience for this paper includes field workers within development organizations and activists working with human rights and health issues at brick kilns, as well as government policy makers wanting to influence the lives of workers at brick kilns now and in the future.

## Methodology

The research team used a “mixed methods” transdisciplinary approach that applied both quantitative and qualitative methods. Climate forecast data was generated from climate modeling tools, specifically the Climate CHIP/HOTHAPS Soft toolset (Climate CHIP 2016). A qualitative analysis was conducted using a transdisciplinary literature review in order to provide the national context. Local case study data was collected around Chennai, India. This included occupational heat stress measurements and observations from brick kilns. A case study research methodology is suitable given the multifaceted ecological and human rights marginalization experienced by people working at brick kilns in India (Flyvberg 2001; Yin 1994). Case study research is concerned with the complexity and particular nature of the case and location in question (Bryman 2008). Chennai was selected as a representative case site where the research question could be applied to both field observations and climate projections so that a transdisciplinary approach could be used to identify potential solutions. The primary field data for the Chennai case study was collected at brick kilns using quantitative methods focused on evaluating occupational health indicators (Venugopal et al. 2016). The case study source materials for qualitative observations were based on a transdisciplinary literature review (Hakim 1987; Kiecolt and Nathan 1985). Transdisciplinarity generally refers to the appropriate combination and integration of knowledge from many different specialties, especially as a mean to shed new light on a problem. The combination of disciplines adds value—the total is more interesting than the sum of the individual contributions or parts (Brewer 1999; Jantsch 1970/72). This requires academic researchers to collaborate across disciplinary, epistemic, and methodological boundaries.

To mobilize the transdisciplinary approach in identifying potential *future pathways*, existing literature was reviewed through a critical discourse analysis lens taking into account the health and environmental risks faced by the people working at the brick kilns. The critical discourse analysis was then directed into a form of framing analysis approach (Fletcher 2009; Jerneck and Olsson 2011; Wise et al. 2014). Framing analysis is a form of discourse analysis. By *discourse*, we apply what social theorist Michel Foucault would describe as “a way of talking about or dealing with a phenomenon” (Ingstad and Whyte 1995). Thus, the Foucauldian notion of discourse is a culturally constructed representation of reality that is used to construct knowledge and thus governs, through the production of categories of knowledge (including disciplinarily categories) and assemblages of texts, what it is possible to talk about and what is not (Foucault 1972/1995; Foucault 1991).

Framing analysis exposes the role of “political language and worldviews in the construction of plausible, meaningful and socially relevant pathways that can enroll a majority of stakeholders and citizens in collective action” (Fletcher 2009). Taking a solutions-oriented approach to framing analysis, we applied discourse analysis as a way of showing “how problems are embedded in particular narratives.” Further, an applied approach was taken to illustrate how “reframing stimulates alternative understandings and problem solutions” by applying the capacity of reframing to associate ideas across theoretical, empirical, and disciplinary divides as a way to break through “disciplinary compartmentalization” traps that ignore the complexity of cross-cutting sustainability challenges (Jerneck and Olsson 2011).

Identifying new solution pathways is critical for saving lives as the risks posed by climate change grows. Studies have pointed out that “the nature and effectiveness of responses” to climate change are strongly influenced by framing (Wise et al. 2014).To realize the solutions-driven focus of this study, potential future pathways were uncovered discursively in a step-wise approach that applied *framing analysis as method*. The first step was the transdisciplinary literature review. Migrant brick kiln workers in India face heat risks from multiple causes that require a holistic analysis. Due to this, the authors found it a critical workplace to study and conduct a transdisciplinary analysis. The literature review was transdisciplinary because the researchers intermixed a diverse range of disciplines, including environmental science and engineering, architecture, human ecology, social anthropology, and occupational health. The analysis could have included more disciplinary perspectives; however, this was not possible due to time and other structural restrictions. The disciplinary frame brought into the study by each researcher was leveraged to evaluate the discourse uncovered in the literature review. The second step was to engage in an initial deep interactive discussion about the literature as a way to identify both synergies and incongruences based on disciplinary perspectives and empirical data. The third step was a final reframing discussion during which the discourse uncovered in the literature review was analyzed together with the case study results combined with challenges imposed by climate change-induced heat. Additionally, the overall challenge in the reframing analysis was to uncover ways of providing low-cost, soft solutions accessible and attainable at low cost for local people whenever possible.

## Research findings: current state

### Transdisciplinary literature review: climate change reinforcing heat feedback loops

Brick kilns in India produce between 150 and 200 billion bricks annually (CATF 2012), a building material that has been predominantly used during India’s construction boom. The brick kilns only stop operating during the rainy season, so the brick industry relies on largely manual labor throughout the dry season and is generally confined to rural and peri-urban areas.

The Central Pollution Control Board has identified brick making as one of the most polluting industries in the small-scale sector (Development for Alternatives 2012).Bricks are commonly fired to a temperature of 700–1100 °C, requiring an enormous amount of energy for the firing operation, resulting in brick kiln owners sourcing fuel from many supply streams. The pollution generated as by-products of the combustion process can generate significant negative local health impacts from particulate matter, black carbon, sulfur dioxide, oxides of nitrogen, carbon monoxide (CO), and black carbon/soot that contribute to worsening of climate change globally (Menon et al. 2002). Black carbon by itself is a major health hazard (Löndahl et al. 2010), so even mitigating soot production alone could significantly reduce the rate of warming over the next decades (UNEP/WMO 2011). Brick kilns are estimated to consume roughly 25 million tons of coal per year, thus making them among the highest industrial consumers of coal in the country, which contributes to both climate change globally and local health risks arising from respiratory ailments (CATF 2012; UNEP/WMO 2011). In addition to the brick industry in India consuming an estimated 8% of the total coal consumption in the country, it also consumes a large amount of other fuels, mainly wood and agricultural waste. The industry also uses other available cheap and polluting waste such as rubber tires (Development for Alternatives 2012).

While the technical side of brick manufacturing in India has been covered widely in scientific literature, this has not been the case to a similar extent as regards the human part of the industry. The situation for India’s brick kiln workers, however, gained international media coverage starting in 2014 through a number of spectacular articles and TV presentations. Headlines included “More than two million people work in the *brick kilns* that supply the India’s booming construction sector, and many are held in conditions little better than slavery” (BBC 2014); “Blood Bricks: How Indian urban boom is built on slave labour” (The Guardian 2014); and “India’s booming cities built from ‘blood bricks’ of bonded laborers” (Reuters 2016). Media thus directly combined what they saw as slave-like conditions of brick laborers and their families with India’s growing economy and booming urban construction sector. Here we see a strong image combining the “new” and “old,” the hard and the soft India through the figure of the brick and the person producing it. They were, thus, put into new frames and while the industry is seen to look for hard solutions, the human side, as we argue in this article, needs soft solutions and structural change.

Traditionally, India is a country where people have predominantly lived from agriculture with the local village as the center of existence, providing livelihood directly from the land. The traditional agricultural system was based on farmers’ own-resource-based subsistence farming. With the green revolution, initiated already in the 1960s, this has increasingly moved to purchased input-based intensive commercial farming based on cash crops. After structural adjustment in the early 1990s, and the neoliberal reforms, there has been an increasing marginalization of land holdings together with loss of subsistence farming practices. It is further found that opportunities for non-farm local work in rural areas have declined after the economic reforms with the consequence that employment for rural labor now has a tendency towards relatively more insecure and casual work while secure jobs or self-employment has declined (Reddy and Srijit 2012). This has also created a decline in livelihood opportunities for landless agricultural laborers, thus forcing former peasants into the semi-urban brick kilns (The New York Times 2007).

The main raw materials used for brick kiln production are soil and coal. A study from Bihar (Development for Alternatives 2012) showed that as much as 90% of the soil used for brick production in the state was procured from agricultural land, and if kept in agricultural production, this land could have produced 7000 t of rice, enough to keep 110,000 people with food grain. Given that a large portion of the brick kiln workers in large cities, such as Chennai, migrate from Bihar, one of the poorest states, it indicates another one of the tragic loops that drives the system. Black carbon/soot still furthers the negative loops and necessitating migration as it heats up the atmosphere and thus accelerates the melting of Himalayan glaciers that feed India’s large rivers (Menon et al. 2002; Xu 2009).

To this comes climate change, creating an added environmental crisis of agriculture with rising temperatures, resulting in weather extremes with water scarcity and severe drought in many areas. Further extremes are increasing precipitation with tropical cyclones. This results in greater instability in food production (Aggarwal 2008; Reddy and Srijit 2012). The extension of the technology of the Green Revolution to regions where it was not suitable, such as dry and rain-fed parts of the country, further aggravates the water crisis. Seasonal migration thus becomes a survival strategy for rural people now completely dependent on diminishing land holdings, under increasing environmental stress, as a main source of livelihood for continued life in the village when other opportunities for rural work are disappearing.

Most brick kiln workers do, however, maintain their linkage to their original place of residence, engaging in a form of circular migration where farm work and unorganized work at the brick kiln supplement each other as sources of livelihood for rural poor such as landless laborers and marginal farmers. They migrate for the brick kilns during the dry season when there is not much work in agriculture and return to their villages at the start of the monsoon. They are contracted to the kiln industry in their villages by a local contractor who will know which people are in need of loans and extra livelihood and thus willing to migrate. The contractor gets a commission from the number of bricks made by the worker supplied by him (Molankal 2008). The lure is the lump sum the worker gets in advance and which the family can use for paying back debts and buying foodstuff during an otherwise lean period (Molankal 2008). He and any accompanying family are then bonded to work in order to pay back the advance.

### Local case study field observation: occupational heat stress data from brick kilns near Chennai, India

The quantitative data collection for the case study took place at brick kilns in the local area near Chennai, India. Chennai, the capital city of Tamil Nadu, is located on the Coromandel Coast of the Bay of Bengal and has a hot and humid climate. Chennai is a major administrative and cultural center and has also become one of the major outsourcing destinations within India (Kobayashi-Hillary 2005). Large-scale brick production on the outskirts of Chennai dates back to the mid 1970s and the state is one of India’s most important brick-making states due to strong growth in the construction sector (Prakash 2009). Further, it has been backed by a vast housing scheme from the Tamil Nadu Government that was unaffected by the global economic crisis of 2008 (Guérin et al. 2012).

The field study focused on gathering occupational heat exposure in order to understand the health risks faced by people working at brick kilns. It confirmed that brick kilns are work sites where people are exposed to both extreme outdoor temperatures (maximum ranges between 40 and 45 °C in the hot months) and high radiant heat from brick kiln furnaces over many hours (globe temperatures of between 19 and 52 °C year-around). Additionally, high levels of heat stress are experienced by people working at the brick kilns due to the limited or non-existent cooling options provided on-site by brick kiln owners.

#### Environmental heat measurements

An assessment of the thermal environment was conducted in accordance with occupational health and safety guidelines issued by the International Labour Office (ILO) and the International Organization for Standardization (ISO). The ILO guidelines state that “measurements of thermal conditions should take account of: (a) all stages of work cycles and the range of temperature and humidity under which the tasks are performed; (b) the range of clothing worn during the tasks; (c) major changes in physical activity level (metabolic heat production)” (ISO 1989; ILO 2001). Measurement techniques include the use of the Wet Bulb Globe Temperature (WBGT) index (ILO 2001). The WBGT index is a common occupational health-screening tool used for setting limit values for work in both indoor and outdoor environments (Bernard et al. 2005; Gao et al. 2017 this issue; Parsons 2014).

The ISO 7243 standard incorporates environmental temperature, humidity, and solar radiation (ISO 1989). Air temperature (*T*_*a*_), natural wet bulb temperature (*T*_*nw*_), and globe temperature (*T*_*g*_) were collected using the 3M™ QUESTemp°™ 32 heat stress meter (accuracy of *T*_*a*_, *T*_*nw*_, and *T*_*g*_; ± 0.5 °C and *RH*; ± 5%) during the colder (January–February) and warmer (March–July) months. The QUESTemp uses a 5-cm diameter globe for a faster response time while the WBGT index is based on the response of a 15-cm diameter globe. As a result, the instrument correlates it to match that of a 15-cm globe. Average WBGT values and minimum and maximum values (Table 1) were measured at five brick kilns: one from Salem, Trichy, Chengalpattu, and two from Thiruvallur district. Due to access constraints from employers and other practical issues, measurements were not possible throughout the day or for a number of days. Therefore, the measurements are averages from different work sections in each workplace for between 3 and 6 h.

**Table 1:** Seasonal comparative table—WBGT summary measurements from five brick kilns in the Chennai area (2013–2015)

		WBGT min	WBGT max	WBGT average
Hot season	June–July 2013	25.8	28.6	27.0
	March–April 2014	24.8	35.0	29.0
Cool season	February 2013	18.8	25.2	22.2
	January–February 2015	22.9	28.3	25.6

The brick kilns have high heat exposure in the hot months (see maximum values in Table 1 and the month of May in Table 2), with WBGT sometimes exceeding the international standard limit value of 28 °C for acclimatized workers under moderate workload (ISO 1989).

**Table 2:** Example brick kiln from Chennai in 2013—seasonal comparative table with data from different work stations

Work location	May			January		
	Temperature (°C)	Humidity RH (%)	WBGT (°C)	Temperature (°C)	Humidity RH (%)	WBGT (°C)
Brick production from clay	29.3	57	25.9	20.0	88	18.8
Brickmaking area	30.5	33.0	27.8	22.0	79	25.2
Furnace area 1	27.0	28.9	25.8	20.0	88	20.5
Furnace area near chimney	27.0	28.9	26.2	20.5	88	20.7
Furnace area 2	27.0	28.9	26.6	20.0	88	21.0
Furnace area 3	27.5	29.5	26.6	20.0	88	21.4
Furnace area 4	30.5	33.0	28.6	22.0	79	24.9
Working place 1	30.0	48.5	25.6	20.5	88	22.4
Brick loading area	28.0	30.2	26.0	22.0	79	24.7
Average WBGT	26.7 (SD: 1.0)			22.2 (SD: 2.1)		

#### Internal heat production

Most workers in the Chennai brick kiln had moderate workloads (130–200 W/m^2^; ISO8996 2004), all in direct sun exposure (Lundgren et al. 2014). From brickwork simulations with 1.7-kg bricks carried out by the authors in a climate-controlled chamber measuring oxygen consumption, the average metabolic rate was 133 W/m^2^ (SD 15 W/m^2^). From measurements from 114 brick kiln workers in the field of between 3 and 6 h, heart rate averaged 87 bpm in the cool season and 88 bpm in the warm season with a maximum of 116 bpm and a minimum of 60 bpm. Sweat rates averaged 0.4 l/h in the cool season and 0.8 l/h in the warm season with a maximum of 1.2 l/h.

From field observations, the main work tasks have varied workloads following these steps:

1. *Material Procurement*: The clay is mined and stored in the open.
2. *Tempering*: Clay is mixed with water to get the right consistency for molding. Mixing is done manually with hands and feet.
3. *Molding*: A lump of mix is taken, rolled in sand, and slapped into the metal mold.
4. *Drying*: The bricks are emptied onto the drying area, where they are dried in the sun. Every 2 days, they are turned over to facilitate uniform drying.
5. *Firing*: The bricks are arranged in a kiln and insulation is provided with a mud-pack. Fire holes are ignited and the kiln is later sealed to keep the heat inside.
6. *Sorting*: After the kiln is disassembled, the bricks are sorted according to color. Color is an indication of the level of burning.

#### Work clothing trapping heat

Clothing also affects the level of heat exposure for each person (ILO 2001). Traditional clothing such as churidars and saris are commonly worn at the kilns by women, with an average clothing basic insulation range of 0.58 clo for the churidar to 0.96 clo for a sari with a protective shirt and towel on the head. Men usually wear a shirt and trousers with an average clothing insulation of 0.61 clo (Lundgren et al. 2014; Havenith et al. 2015). The sari has also proven to be a very effective garment to protect workers from heat (Indraganti et al. 2015); however, its effectiveness is hampered by the protective shirt (Lundgren et al. 2014). Indirectly accounting for moderate metabolic rate, a work limit value for WBGT would be around 28 degrees for the acclimatized worker (ISO 7243 1989). Further, in the present study, clothing adds further heat stress in particular for females.

#### Self-reported heat stress

Questionnaire data with simple yes and no questions regarding health and productivity was also collected at the brick kilns in Chennai and the results are presented in Table 3.

**Table 3:** Comparative table depicting self-reported impacts of heat stress on health and productivity from a questionnaire (*N* = reported cases)

Variable	Impacts	Summer (*N* = 87) June–July 2013 and March–April 2014		Winter (*N* = 61) February 2013 and January-February 2015	
		*N*	Percent (%)	*N*	Percent (%)
Impacts on health	Excessive sweating	82	94	40	66
	Muscle cramps	32	37	28	46
	Tiredness/weakness/dizziness	75	86	44	72
	Headache	55	67	19	31
	Nausea/vomiting	7	8	3	5
	Fainting	13	15	6	10
	Prickly heat	21	24	7	11
Production target and issues	Have production target	2	2	10	16
	Able to complete production target	2	2	9	15
	Not able to complete production	–	–	1	2
Impacts on productivity	Absenteeism/taken sick leave due to heat	14	16	10	16
	Less productivity/more time to complete task/work extra hours	42	48	10	16
	Irritation/interpersonal issues	16	18	1	2
	Wages lost	–	–	5	8
Coping mechanisms	Take rest	65	75	60	98
	Drink water	87	100	61	100
	Cool shower, bath, or sponge bath	24	28	1	2
	Traditional methods (e.g., drinks and diet, self-pacing)	49	56	14	23
Impact of clothing on comfort	No impact	72	83	60	98
Impact of clothing on productivity	Moderate impact	24	28	12	14
	High impact	4	5	1	1

These results are consistent with concerns raised about the harsh working conditions found at brick kilns. Most workers report symptoms of heat strain, reduced productivity, and impacts on daily life during the hot season, which lasts for over 6–8 months. However, a bias could have been introduced in the collected data as the employer was always present during the questionnaire.

## Analytical discussion: reframing potential future pathways

This analytical section uses framing analysis as a way forward to identify “pathways” for change. This qualitative way of framing solutions borrows the “pathways thinking” metaphor used in the sustainable development discourse (Wise et al. 2014).

### The climate connection: forecasted climate change-related heat impacts

**Fig. 1:**
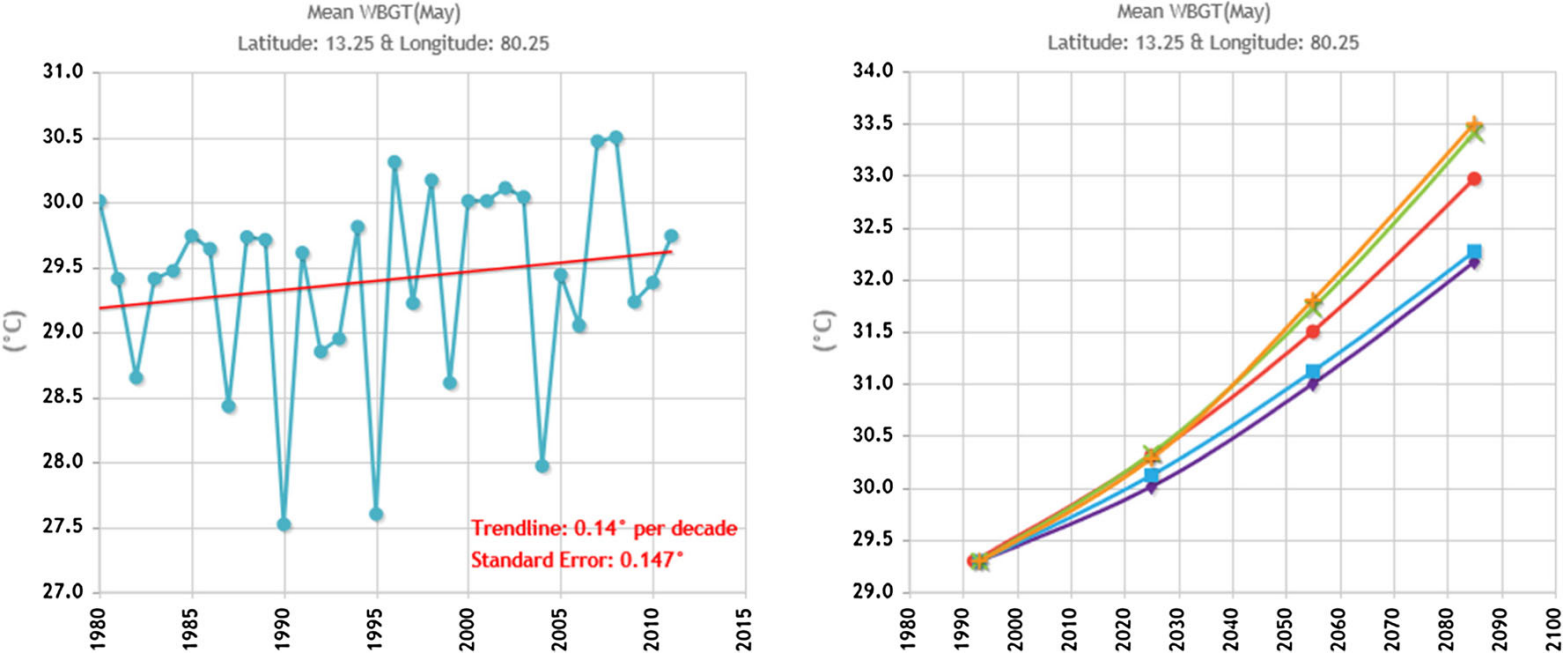
Historical and future heat stress during the month of May in Chennai, without taking solar radiation into account, according to measurements and simulations. Produced by HOTHAPS soft (Kjellström et al. 2013; Lemke and Kjellström 2012; Climate CHIP 2016). The different colors represent different models datasets of RCP 8.5 (, , , , and )

India is already at high risk of excessive heat. In many places, the maximum temperatures during some parts of the year already exceed 40 °C. An additional 3–5 °C will make outdoor physical work very difficult during the hottest periods (Kjellstrom 2009; Venugopal et al. 2016). Figure 1 shows the average heat stress index WBGT (ISO 1989) during the month of May in Chennai over time and future modeling based on the IPCC’s representative concentration pathway of 8.5 (Climate CHIP 2016; IPCC 2013). The WBGT is calculated from airport weather station data and modeling data from the University of East Anglia, UK, produced by HOTHAPS Soft (Kjellström et al. 2013; Lemke and Kjellström 2012). Hence, solar radiation is not accounted for in this data set. WBGT levels are already close to or even above the limit values identified for brick kiln work in Chennai (specifically a WBGT of 28 °C), and that is without even accounting for solar radiation and the contribution of the urban heat island effect (Kleerekoper et al. 2012). Temperatures are projected to climb above the limit values of 33 °C for a resting acclimatized worker by the end of the century (Fig. 1), thus worsening conditions in Chennai.

Given these potential thermal futures, the need for radical interventions becomes even more apparent. Our analysis now turns to ways of redefining the problems and finding new soft solutions to overcome the complex challenges faced by people working at brick kilns.

#### Reframing the role of technology: from high tech to “appropriate technology”

It became apparent during the literature review that experts have tended to focus primarily on technical solutions whether using infrastructure changes such as improved smoke stacks, or fuel mix modifications at the kiln furnace. Furthermore, the solutions suggested tend to focus on mitigating problems affecting populations living far away from the brick kilns, but not specifically on health challenges facing the local migrant populations working at the brick kilns to frequently live either on-site or nearby the facility. For example, in the Clean Air Task Force’s 2012 report on brick kilns, the final roadmap includes many technical solutions, but only in the final sentence of its recommendations do they suggest their technical solutions may lead to an “improvement of working conditions for millions of workers employed in brick kilns” (CATF 2012: XXIV).

In this next section, “appropriate technologies” are suggested that may actually be better suited to resolving some of the heat-related social and environmental issues affecting people working at brick kilns.

### Locally “appropriate technology” approaches

Alternative choices in building materials could minimize environmental hazards and reduce the health impacts of brick production. One alternative is to use sun-dried mud bricks as a more locally appropriate technology. In Chennai today during the working season, the sun provides a free and abundant source of heat. Thus, the sun can be used as a natural fuel for drying the bricks. Experiments with using sun-dried earth bricks (Fathy 1973; Kennedy 2004) show that using local and natural earth materials in buildings is energy efficient, low in toxicity, safe, and durable, especially if obtained from the local environment. Other research shows that using low energy-intensive earth building materials could be an asset in reducing CO_2_ emissions as well (May 2010; Rael 2009). The use of locally sourced materials can provide social and economic benefits locally while also reducing production costs compared to using both imported and industrialized building methods and materials (Morela et al. 2001). In countries where labor is abundant and labor costs are low, using the existing workforce of craftsmen and skilled locals opens up more job opportunities for local people (Dabaieh 2011) while strengthening the local community and reducing the dependency on energy-consuming construction techniques.

Another benefit of using mud brick and other locally produced natural building materials is the ability to ensure building materials are locally produced, recycled, and re-used, as well as being simply able to return to Earth as soil for vegetation (Morela et al. 2001). The sun-dried bricks can be promoted in the local market as an environmentally friendly building material, and help local consumers to gradually become aware of “green” issues. Legislation can help introduce sun-dried bricks into the marketplace and put into effect environmentally friendly building practices. The bricks also have significant implications for long-term costs, building performance, and energy consumption due to their thermal properties. Using sun-dried clay brick can help in producing so-called zero carbon buildings for two reasons. Firstly, the embodied carbon from energy usage during brick production is minimal thanks to using sunlight instead of coal or firewood in the drying process. Secondly, the sun-dried bricks can become CO_2_ storage sinks if lime is added to the clay mixture of the bricks during production to help the bricks take CO_2_ out of the air. Basically, the lime acts to provide this CO_2_-absorbing capability by changing the physical properties of the clay to improve the water resistivity properties of the bricks (Jones 2005). Plastering sun-dried brick walls with lime plaster then rendered with casein protects the external brick surface from water erosion during rainy seasons. However, one caveat is that the weather in Chennai can be quite humid. Even though the lime needs humidity for the complete chemical process in the clay mix to take place, the humidity may impair the sun-drying process.

Local initiatives have already begun trying to pilot projects using cast mud bricks in building construction (Auroville Earth Institute 2016). The know-how is there, as is the tremendous potential to gradually transform brick manufacturing into a cleaner industry. If sun-dried mud bricks are cast manually, one person can cast up to 700 bricks (25 × 13 × 12 cm) in 8 working hours. If compared economically with fuel prices, sun-dried mud brick production could be far more economical, thus making the final brick price more competitive in the market. Further, the burning process takes almost the same time as it takes for the sun-dried bricks to dry in the sun. From a life-cycle analysis and cradle-to-cradle perspective, the embodied energy in creating the sun-dried mud bricks, from manufacturing to demolition and reuse, is minimal. Thus, sun-dried bricks have the potential to address more challenges overall compared to the other existing brick-manufacturing options available today, such as fly ash bricks, perforated bricks, and hollow cement bricks. Retrofitting the existing brick factories could take advantage of their mixing and casting infrastructure, while removing the energy intensive, heavily polluting firing process found in India’s current brick kiln factories.

#### Reframing the role of people: from global human capital to local human rights

A recent UN report on the connection between labor and climate change argues for incremental interventions that focus on policy recommendations that emphasize the use of direct occupational health approaches that look to ILO guidelines (UNDP 2016), but only under business-as-usual structural regimes. The report mentions “productivity” 107 times, while never once recommending a rights-based approach for protecting people and the environment from a changing climate. The report does not evaluate the risks of coercive bonded labor practices, inequality, and oppression increasing among vulnerable populations who are forced to work at sites where heat exposure is already high and will worsen under climate change, as could happen with people working at brick kilns. However, the ILO stated as early as 2005 that brick kiln work “is a particularly prominent feature of contemporary forced labour situations” (Srivastava 2005). This has been corroborated by other reports that have pointed out that industrial brick production sites have been found to be sites of neo-bondage, mediated by middlemen posing as recruitment agencies (Breman 1996, 2007; Molankal 2008).This underscores the need to reframe the discussion from being about how to maximize the productivity of global human capital to being about a rights-based approach to protecting people and the environment. In this next section, we present framings that incorporate localization strategies, and an approach for protecting environmental sustainability along with local human rights.

### Participatory socio-culturally informed localization approaches

Loss of local decision-making influence can be a side effect of globalization. Localization helps local people bridge the growing democratic influence gap by regaining influence within their communities. Localization of decision-making could provide a way to regain self-sufficiency for India’s village communities. Using an open participatory process could also engage a wider range of local actors, including vulnerable groups such as migrant workers. Re-localization could be described as a process in which “people are reaffirming control of their lives” (by) putting culture and dialog back at the heart of their efforts to liberate society (Liegey et al. 2015). It is not too late to reach for what Metcalfe (1833) recommended, which was to cultivate India’s local village economies, which were later called village republics and village *swaraj* by M. K. Gandhi (Mandelbaum 1970).The Delhi-based Centre for Science and Environment similarly argued for decentralized governance and sustainable village ecology as an answer to rural India’s threatened ecological existence. In their 1995 video *The Village Republic*, which followed up on their 1989 landmark publication, *Towards Green Villages*: *A Strategy for Environmentally*-*Sound and Participatory Rural Development*, they reported on “pioneering community-based rural natural resource regeneration efforts in India carried out in the 1970s and 1980s” as practical examples of the Gandhian “Village Republics” concept (CSE 2017).

Then there is the open re-localization approach, which emphasizes “openness” in order to underscore the collaborative and dialog-based dimensions of the localization process beyond nationality or religion, so as to instead celebrate diversity and provide “democratic tools that facilitate dialogue-driven, power sharing community arrangement[s] that pave the way towards non-violent outcomes that protect human rights and ecological sustainability” (Liegey et al. 2015). This shift to local economic decision-making would help local communities to regain control of what they produce and exchange through Social and Solidarity Economy (SSE) practices. SSE is used by organizations that are actively pursuing “social aims and fostering solidarity” (ILO 2014), and provides a way for local individuals and organizations to benefit from economies of scale and reduce costs, and achieve a common goal that would otherwise be unreachable individually (ILO 2002).

SSE also builds on ideas Peter Kropotkin described as “mutual support” in his book *Mutual Aid*: *A Factor of Evolution*, originally published in 1902, in which he declared that “mutual support – not mutual struggle – has had the leading part” in shaping what could be called “progress” in human communities as a counterpoint to the social Darwinism rooted in competition (Kropotkin 2014). Bringing local people into a participatory process to define both the problem and solution spaces would be key anchors in a process of “rehabilitating the political realm [by putting] culture and dialogue back at the heart of our efforts to liberate society [and] actively calling into question [...] the primacy of the economy and of work as society’s central values” (Liegey et al. 2015) so that socio-culturally informed ideas from the people themselves could enter the deliberative decision-making process. This would help migrants to begin to gain power over their own lives and to escape the neo-bondage.

### The environmental sustainability and human rights-based approach framework

The most fundamental issue facing the people working at brick kilns is the system that perpetuates brick kilns as sites of coercive bondage and neo-bondage, which constitutes nothing less than institutionalized slavery. Addressing heat and climate change without considering human rights and ecological injustices ignores the obvious “elephant in the room” when it comes to addressing brick kilns in a broader, socio-culturally informed fashion. Based on the evidence found at the Chennai field site and the climate change projections from the climate model, the hazards facing the migrant labor population are undeniable. The technical solutions presented in the previous section provide one pathway towards reconciling the human health threats facing the local people. Thus, this section details a socially inclusive pathway towards change. This socially inclusive perspective is used to reframe the solution space in which inclusive solutions are formed. Rather than formulating a top-down strategy, this section suggests a way in which solutions could be co-created alongside local populations as part of inclusive, iterative participatory processes. This more inclusive bottom-up approach provides a way for local people, including migrant populations, to become involved in work with local development agents to reframe the issue in a way that integrates considerations that reveal linkages between human rights violations and ecological injustice. Taking this approach provides a venue for raising climate change mitigation and adaptation strategies, while also providing an approach through which a new pathway can be formulated to reveal the true benefits of “what development is [meant] to achieve” (GI-ESCR 2014).

The Global Initiative for Economic, Social and Cultural Rights (GI-ESCR) has published training materials for international development projects, advising an approach to rolling out development projects in order to secure “human rights for the current generation within a sustainable amount of ecological space that does not compromise the human rights of future generations” (GI-ESCR 2014). By emphasizing the need to integrate human rights and environmental sustainability as the core success criteria for sustainable development, the GI-ESCR has created what could be called the environmental sustainability and human rights-based approach (ES-HRbA) framework. The framework described in the guide is intended for application at the local level (GI-ESCR 2014), which also gives it the potential to be a critical tool when evaluating pathways for change. It provides a powerful framework of analysis and a basis for action, while helping to understand and guide development at the local level (GI-ESCR 2014).

The first stage of the ES-HRbA is to analyze the underlying causes of social injustice. Using environmental justice theory as the underlying framing in ES-HRbA provides a tool to “examine how *social injustice* is inextricably bound up in *ecological injustice*” by examining how discrimination and marginalization of vulnerable groups manifests. In the case of this study, the vulnerable groups we are referring to are workers at brick kilns’ sites (Breman 1996, 2007; Molankal 2008). Particularly from an environmental justice perspective, brick kiln workers also become vulnerable by virtue of living and working where pollution and hazard intersect with poverty and exclusion, coining the terms “environmental discrimination” (GI-ESCR 2014). Interventions can then be designed that focus on local sustainable development strategies for addressing “all humans” needs and capabilities, rather than (focusing on) the accumulation of wealth. Instead, human rights and respect for ecological boundaries become the focus (GI-ESCR 2014).

The next stage of the ES-HRbA combines the fight for human rights with the need for subsistence rights through resource conservation. This would help marginalized rural populations to escape or avoid farm debts by securing subsistence rights to control natural resources. Protecting subsistence rights as “a central plank of natural and environmental conservation [helps ensure that] resources of the poor will not be easily diverted to the rich” (Sachs 2003).

By focusing on human rights at the brick kilns in India, they cease to be just another kind of workplace with a voluntary workforce. The human rights focus would demand the immediate abolition of all forms of slavery, including bondage and neo-bondage, in accordance with both international laws against human trafficking, as well as the federal laws in India, which are criticized for being seldom enforced. Finally, there would be a clear mechanism for national and state authorities to enforce existing international laws from the UN and the ILO, in addition to existing national laws already ratified in India prohibiting child labor, bonded labor, and neo-bondage. This step alone could protect people from extreme heat exposure by addressing the enslavement of thousands of rural people, including migrant adults and their children in forced labor conditions, within India’s brick kiln factories.

#### Limitations to our analysis

Limitations to our analysis include a lack of time and resources to pilot solutions through actual interventions in the field. This is something that creates a future opportunity for new research, and practical interventions, whether through local engagement using action research or through federal level interventions. While differences in responsibility and impacts were reviewed between countries, as well as within India between the rural-urban population, this study did not investigate solutions to curtail the contribution of India’s affluent population to climate change. India’s growing affluent population shoulders some of the responsibility for climate change-inducing emissions, whether as brick kiln business owners or as consumers of energy-intensive products and services coming from the fossil fuel intensive economy. Thomas Piketty and Lucas Chancel describe the inequality of energy intensity usage measured as carbon dioxide equivalent (CO2eq) emissions within every country in their 2015 report. Their report stated that “within-country inequality in CO2eq emissions matters more and more to explain the global dispersion of CO2eq emissions [so] it is then crucial to focus on high individual emitters rather than high emitting countries [since] within-country inequality [now] makes up 50% of the global dispersion of CO2eq emissions” compared to the one third it accounted for in 1998 (Chancel and Piketty 2015). Thus, the rapid growth of emissions from India’s affluent should be examined critically.

## Conclusion

Climate change-induced heat drives feedback loops that increase hardships for workers at brick kilns in Chennai on multiple fronts. The increasing heat at already hot brick kilns in Chennai brings forward a multi-dimensional issue that demands first and foremost a pathway towards addressing the egregious human rights violations at the brick kilns, while at the same time also ensuring the reduction of pollution through locally appropriate technical solutions. Brick kilns around Chennai are already reaching the limits for occupational heat exposure and are also sites of institutionalized slavery. We argue that it is necessary to take a holistic approach in order to ensure that solutions for people living in and around brick kilns will help them to make a transition that includes a mix of locally appropriate technologies and human rights-based approaches. This could lead to regulatory interventions that are based on locally defined socio-cultural priorities that speed structural shifts towards environmentally sustainable, human rights-based outcomes.

The federal government can create structural conditions that either further privilege the affluent urban populations in accumulating more wealth through more globalization as was done with the SAPs, or assist the rural populations to regain their former resilience through more localization. We focused on the possibility for more domestic, local interventions, since it is on the local level that populations will need to face the looming climate crisis.
